# Out of the ESCPE room: Emerging roles of endosomal SNX‐BARs in receptor transport and host–pathogen interaction

**DOI:** 10.1111/tra.12885

**Published:** 2023-04-23

**Authors:** Boris Simonetti, James L. Daly, Peter J. Cullen

**Affiliations:** ^1^ Charles River Laboratories, Discovery House, Quays Office Park Conference Avenue, Portishead Bristol UK; ^2^ Department of Infectious Diseases School of Immunology and Microbial Sciences, Guy's Hospital, King's College London London UK; ^3^ School of Biochemistry, Faculty of Life Sciences, Biomedical Sciences Building University of Bristol Bristol UK

**Keywords:** cell biology, endosomes, host–pathogen interactions, infection, lysosomes, membranes, xenophagy

## Abstract

Several functions of the human cell, such as sensing nutrients, cell movement and interaction with the surrounding environment, depend on a myriad of transmembrane proteins and their associated proteins and lipids (collectively termed “cargoes”). To successfully perform their tasks, cargo must be sorted and delivered to the right place, at the right time, and in the right amount. To achieve this, eukaryotic cells have evolved a highly organized sorting platform, the endosomal network. Here, a variety of specialized multiprotein complexes sort cargo into itineraries leading to either their degradation or their recycling to various organelles for further rounds of reuse. A key sorting complex is the Endosomal SNX‐BAR Sorting Complex for Promoting Exit (ESCPE‐1) that promotes the recycling of an array of cargos to the plasma membrane and/or the *trans*‐Golgi network. ESCPE‐1 recognizes a hydrophobic‐based sorting motif in numerous cargoes and orchestrates their packaging into tubular carriers that pinch off from the endosome and travel to the target organelle. A wide range of pathogens mimic this sorting motif to hijack ESCPE‐1 transport to promote their invasion and survival within infected cells. In other instances, ESCPE‐1 exerts restrictive functions against pathogens by limiting their replication and infection. In this review, we discuss ESCPE‐1 assembly and functions, with a particular focus on recent advances in the understanding of its role in membrane trafficking, cellular homeostasis and host–pathogen interaction.

## Graphical abstract

Endosomal cargo sorting is an essential process in eukaryotic cells. Here, we review the recently characterized role of the Endosomal SNX‐BAR Sorting Complex for Promoting Exit‐1 (ESCPE‐1) in mediating sequence‐dependent endosomal cargo sorting, its subversion by intracellular pathogens during the infection process, and its restrictive role against a range of viruses.

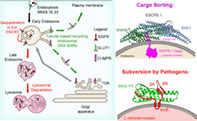

## 1 INTRODUCING ENDOSOMES AND ENDOSOMAL SNX‐BARS

### 1.1 The endolysosomal pathway

**FIGURE 1:**
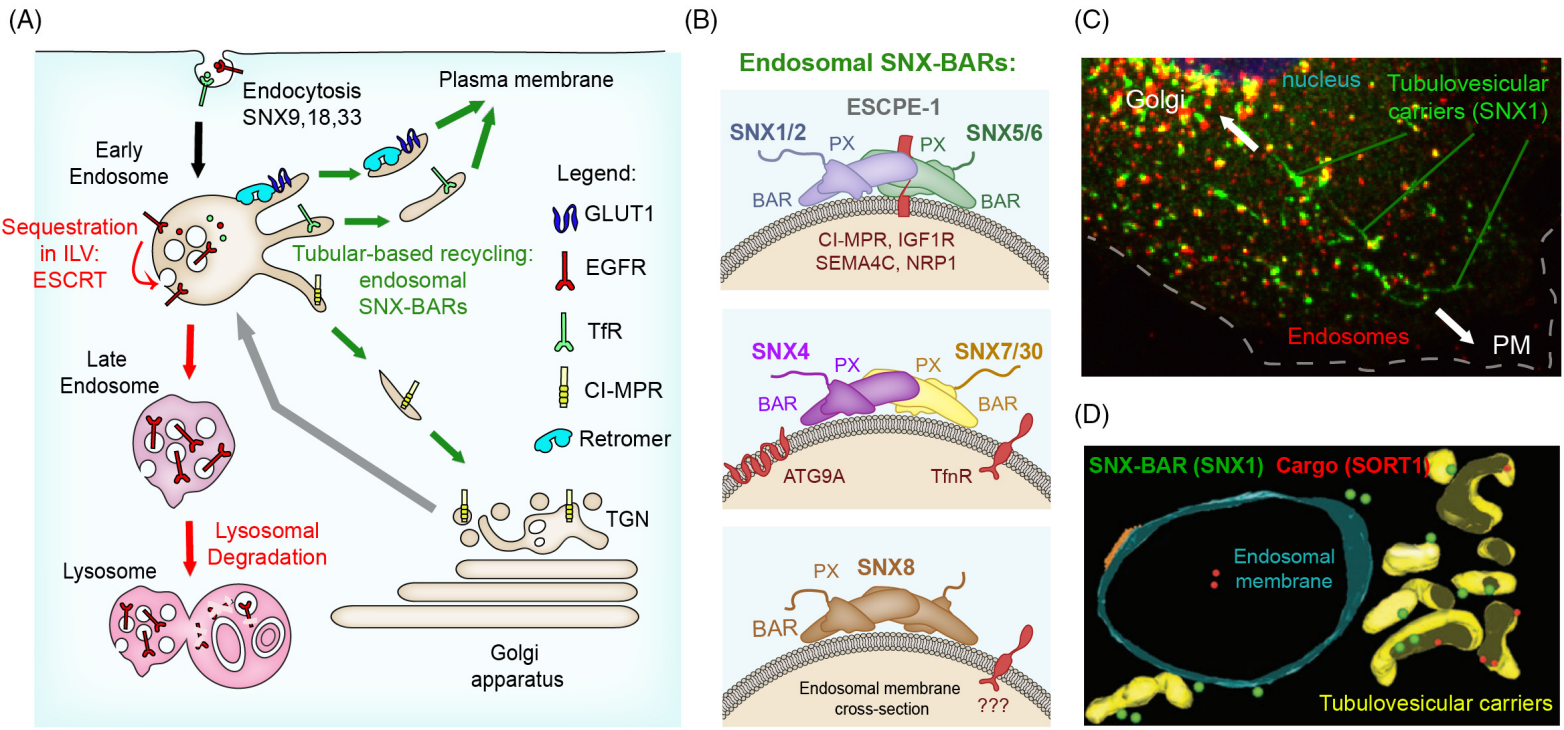
Role of SNX‐BAR proteins in tubular‐based endosomal sorting of transmembrane proteins and associated proteins and lipids (cargoes). (A) Cargoes reach the endosomal system from either the biosynthetic pathway or from the plasma membrane through endocytosis. Within endosomes, cargoes can either be fated for degradation (such as the epidermal growth factor receptor [EGFR]) by the endosomal sorting complex required for transport (ESCRT). Alternatively, cargoes can be retrieved from this fate, and recycled through their sorting into endosomal tubular profiles, from where they are clustered in tubulovesicular transport carriers. These transport carriers can either deliver cargoes to the cell surface (e.g., the Retromer‐dependent Glucose transporter 1 [GLUT1], and the Retromer‐independent Transferrin receptor [TfR]) or to the *trans*‐Golgi network (TGN) (e.g., cation‐independent mannose 6‐phosphate receptor [CI‐MPR]). The family of endosome‐associated SNX‐BARs are among the most important orchestrators of tubular‐based recycling from endosomes. (B) The endosomal SNX‐BAR assemblies that participate in tubular‐based recycling include: ESCPE‐1 consisting of heterodimers of SNX1 or SNX2 with SNX5 or SNX6 (or the neuronal SNX32), heterodimers of SNX4 with SNX7 or SNX30, and SNX8 homodimers. Examples of transmembrane proteins that recycle through these different complexes are depicted in red. (C) Confocal microscopy micrograph of endosomes (in red) from where SNX1‐positive tubular profiles (green) emanate to mediate cargo recycling to the *trans*‐Golgi Network (TGN) or to the plasma membrane (PM). (D) Morphology of the cargo‐enriched SNX1 intermediates examined by three‐dimensional electron tomography. Part figure D has been adapted with permission from the *Traffic* article.^165^

Endosomes are a series of intracellular membrane‐bound compartments that receive transmembrane proteins and associated proteins and lipids (hereinafter referred as to cargoes) from the endocytic and the biosynthetic pathways. The discovery and characterization of endosomes arose from studies exploring Semliki Forest Virus (SFV) entry into hamster kidney cells, establishing their central importance in cellular infection.^1^ The compartments first receiving incoming endocytic material are called “early” or “sorting” endosomes because they represent the primary platform for cargo sorting. Cargo essentially face one of two fates: either they are sorted for entry into lysosomes leading to their degradation, or cargo are retrieved from this fate and promoted for recycling to different cellular organelles^2^ (Figure 1A). Early endosomes undergo a process of maturation into late endosomes by progressively losing cargo via the process of tubular‐based recycling and by accumulating changes in luminal pH and the protein and lipid content of their membrane bilayer.^2^, ^3^, ^4^ When all the recycled material has been removed the late endosome fuses with the lysosome, leading to the formation of a hybrid organelle named endolysosome in which the remaining contents will eventually be degraded.^5^

Cargoes fated for degradation (such as the activated epidermal growth factor receptor, EGFR) are ubiquitinated and sequestered into vesicular structures that invaginate from the endosomal membrane and pinch off in the lumen of the endosomal vacuole to form cargo‐enriched intraluminal vesicles (ILVs)^6^, ^7^ (Figure 1A). The endosomal sorting complex required for transport (ESCRT), a series of multimeric protein complexes, serves to coordinate recognition of ubiquitinated cargo with the membrane remodelling required for ILV biogenesis. ILV biogenesis is an iterative process leading to late endosomes containing multiple ILVs, hence their alternative name of multivesicular bodies (MVBs).^8^, ^9^, ^10^ Late endosomes become competent to fuse with the lysosome, forming a hybrid endolysosome, where the cargo present within the ILVs is degraded through exposure to a series of hydrolyses and lipases present in the lysosomal lumen.^8^, ^9^, ^10^ Endolysosomes are subsequently resolved back to lysosomes to regenerate this degradative compartment.^5^, ^11^ For an extensive review of the mechanistic regulation of the ESCRT pathway we refer the reader to two excellent reviews.^12^, ^13^

Cargo proteins that require retrieval from the lysosomal degradative fate are sorted into branched tubular profiles from where they are packed into tubulo‐vesicular carriers that recycle them to the relevant compartment^2^, ^14^ (Figure 1A). Three main recycling pathways have been described for prototypical cell surface receptors, these include: direct recycling from endosomes to the cell surface (“fast recycling”); transport from sorting endosomes to the perinuclear endocytic recycling compartment (ERC) and then to the cell surface, which can be followed by integrins (“slow recycling”); and transport from sorting endosomes to the *trans*‐Golgi network (TGN) (“retrograde transport”), the route followed by cation‐independent mannose 6‐phosphate receptor (CI‐MPR).^14^ Other cargoes, such as the transferrin receptor (TfR), can use multiple routes to return back to the cell surface depending on several factors, including the kinetics of ligand dissociation, through either the fast route or a slower trafficking through the ERC first.^15^, ^16^ These different recycling pathways are orchestrated by several multiprotein complexes that associate on the endosomal membrane to coordinate the process of cargo selection with the biogenesis of tubule‐vesicular carriers for cargo transport to the target compartment. These complexes included Retromer, Retriever, the CCDC22/CCDC93 and COMMD (CCC) complex, the Arp2/3‐activating WASH complex, and ESCPE‐1. While most of the aforementioned sorting complexes have been widely reviewed,^14^, ^17^, ^18^, ^19^, ^20^ the importance of ESCPE‐1 and other related endosomal ESCPE complexes has only recently been reappraised and this will be the principal focus of this review.

### 1.2 Overview of SNX‐BAR proteins

Sorting nexins (SNXs) are a family of peripheral proteins that localize to endosomal membranes to regulate intracellular trafficking of cargo proteins through a combination of lipid‐binding and protein–protein interactions.^21^ SNXs are all defined by the presence of the phox (PX) domain that binds to phosphoinositide (PI) lipids found in organelle membranes.^21^, ^22^, ^23^, ^24^, ^25^ A subset of SNXs contain a BAR (Bin/Amphiphysin/Rvs) domain in their carboxy‐terminal region and are therefore named SNX‐BARs.^21^, ^24^, ^26^ SNX‐BARs cycle between the cytosol and organelles of the endosomal network through a common mechanism of membrane association that involves simultaneous detection of several membrane properties via their PX and BAR domains, including curvature, lipid identity and cargo density.^24^, ^26^, ^27^, ^28^ Importantly, a discrete series of hydrophobic and charged interactions in the BAR domain dimer interface ensures a limited scheme of homo‐ and hetero‐dimerization, allowing the formation of a restricted number of functional SNX‐BAR dimers.^29^, ^30^, ^31^ These include the plasma membrane (PM)‐associated homodimers of SNX9, SNX18, and SNX33; and the endosome‐localized homodimers of SNX8, heterodimers of SNX4:SNX7 and SNX4:SNX30, and heterodimers of SNX1/SNX2 with SNX5/SNX6/SNX32^30^ (Figure 1B).

SNX1/SNX2 and SNX5/SNX6/SNX32 associate in different dimeric combinations to comprise the endosome‐associated ESCPE‐1 complex, responsible for tubular‐based endosome‐to‐TGN or endosome‐to‐PM recycling of a myriad of cargoes. ESCPE‐1 is the best characterized, and hence prototypical, of the endosomal SNX‐BAR complexes. SNX4 heterodimers and SNX8 homodimers are evolutionarily conserved complexes that localize on endosomes where they generate tubular profiles that are distinct from those of ESCPE‐1^32^, ^33^, ^34^ (Figure 1B). In mammalian cells, SNX4 is involved in the recycling of TfR to the plasma membrane, and in autophagic membrane trafficking^32^, ^35^, ^36^, ^37^ while SNX8 has a largely uncharacterised function (Figure 1B).

## 2 MOLECULAR ASSEMBLY AND FUNCTION OF ESCPE‐1

SNX1 and SNX2 are paralogous proteins with redundant functions, and the same is also true for SNX5 and SNX6 (and its neuronal variant SNX32).^38^, ^39^, ^40^, ^41^, ^42^ SNX1/2 and SNX5/6 are homologs of the yeast Vps5 and Vps17, respectively, that form integral parts of the pentameric retromer complex in fungi.^20^, ^39^, ^40^, ^43^, ^44^ In mammals, these SNX‐BARs can exist as homodimeric combinations of SNX1 or SNX2, or heterodimeric combinations of SNX1 or SNX2 with either SNX5 or SNX6.^30^, ^42^, ^45^ The SNX1/SNX2:SNX5/SNX6 heterodimers have been historically linked with the mammalian subunits of the Retromer complex (VPS26A [or VPS26B]/VPS35/VPS29), and are often referred as to Retromer‐linked or Retromer‐related SNX‐BARs.^14^, ^46^ However, they have also been shown to coordinate the process of membrane remodelling and cargo sorting in a Retromer‐independent fashion and the complex has been named the “Endosomal SNX‐BAR Sorting Complex for Promoting Exit,” or ESCPE‐1^42^, ^47^, ^48^ (Figure 1B).

ESCPE‐1 heterodimers are best characterized for their function in the tubular‐based recycling of several transmembrane proteins from endosomes to the TGN (e.g., CI‐MPR), or to the plasma membrane (e.g., insulin‐like growth factor 1 receptor, IGF1R)^24^, ^42^, ^43^, ^47^, ^48^, ^49^, ^50^, ^51^ (Figure 1A–C). This role was identified through the depletion of ESCPE‐1 subunits, which triggers a marked localisation of CI‐MPR and IGF1R in endosomes, while failing to repopulate the TGN and the cell surface respectively.^42^, ^43^, ^47^, ^48^, ^50^, ^51^ Furthermore, ESCPE‐1 plays a role in the endosomal recycling of Postsynaptic Density 95/Discs Large/Zonula Occludens‐1 (PDZ) binding motif‐containing cargoes through direct interaction with SNX27 and the Retromer complex.^52^, ^53^, ^54^, ^55^ The retromer complex is a trimeric endosomal sorting complex, consisting of subunits VPS26A/B:VPS35:VPS29, that orchestrates the retrieval and recycling of hundreds of transmembrane proteins through association with cargo adaptors such as SNX27.^52^ The direct association between SNX27 and the unstructured N‐terminus of SNX1/SNX2 allows for Retromer cargoes (e.g., Glucose transporter 1, GLUT1 and β2‐Adrenergic receptor, β2AR) to exit endosomes through ESCPE‐1‐positive tubular profiles (Figure 1A). Accordingly, suppression of the ESCPE‐1 subunits leads to the accumulation of Retromer cargoes such as GLUT1 in early endosomes, resulting in reduced levels on the plasma membrane.^52^, ^53^, ^56^, ^57^ Importantly, suppression of ESCPE‐1 does not perturb the recycling of cargoes linked to other sorting complexes such as Retriever and the CCC complex, suggesting a specificity of the sorting of Retromer cargoes into ESCPE‐1 tubules.^17^

This process of tubular export from endosomes, which we will describe in detail in the following paragraphs, is achieved through the ability of the SNX‐BAR dimer to (i) associate to the peripheral endosomal surface, (ii) directly interact with transmembrane proteins, and (iii) locally remodel these membranes into tubulovesicular cargo‐enriched transport carriers that are transported to the target compartment; together termed “co‐incidence detection”^30^, ^48^ (Figure 1C). The generation of cargo‐enriched carriers and their trafficking by ESCPE‐1 mirrors the general functional principles of other coat complexes: (1) cargo is recognized and concentrated on the donor compartment, (2) cargo‐enriched membranes remodel to form vesicular or tubular profiles, (3) scission of these profiles lead to the formation of isolated transport carriers, (4) carriers couple to molecular motors for movement to the target compartment, (5) tethering molecules capture incoming carriers to facilitate SNARE‐mediated fusion with the correct acceptor compartment.^58^ We will explore in detail how the ESCPE‐1 orchestrates each of these steps.

### 2.1 Endosomal targeting by ESCPE‐1

**FIGURE 2:**
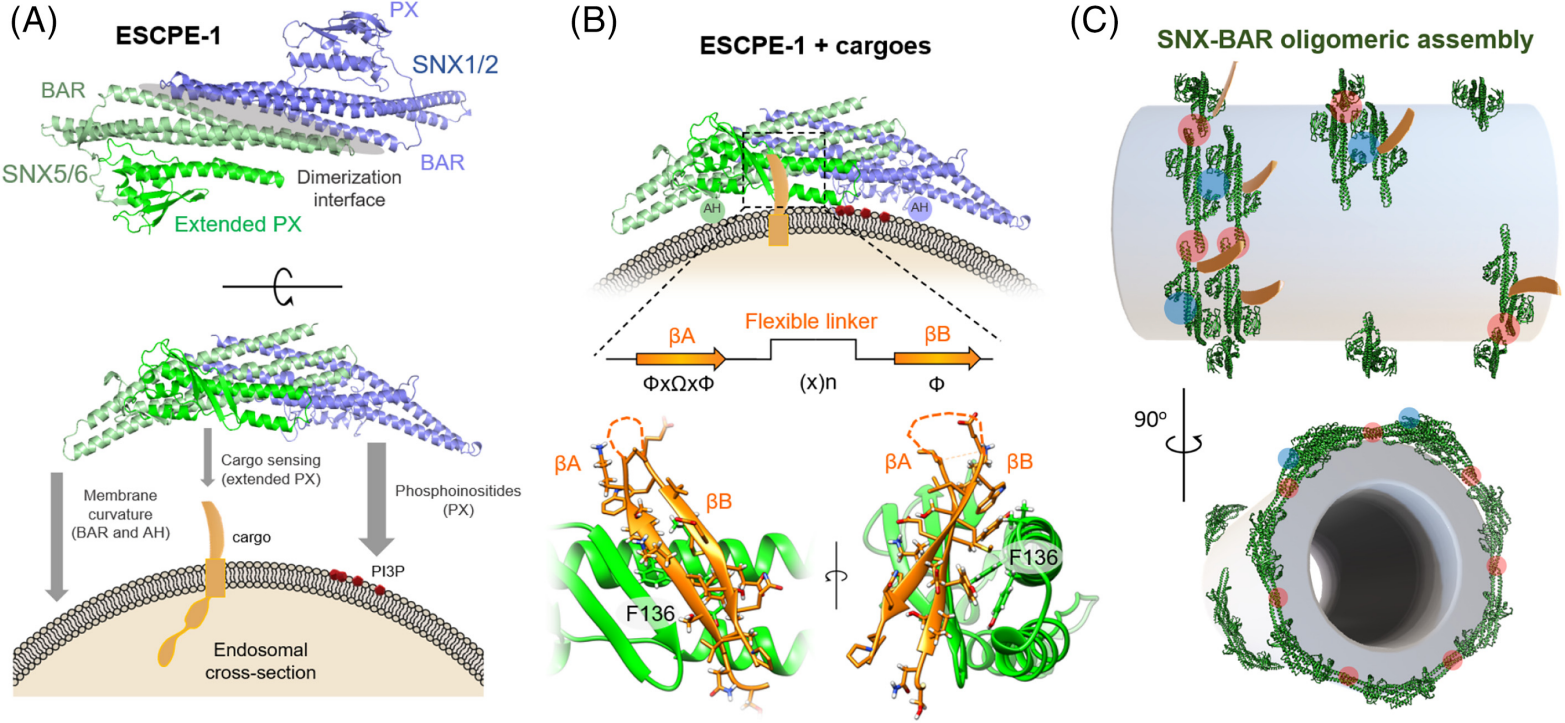
Molecular basis for the biogenesis of ESCPE‐1 transport carriers. (A) Model of the mechanism by which ESCPE‐1 heterodimers sense multiple features of endosomal membranes, including the presence of specific phosphoinositides, local membrane curvature and cytosolic tails of transmembrane proteins. By sensing these features, ESCPE‐1 assembles into functional membrane‐associated complexes that couple membrane remodelling with cargo recognition for the formation of cargo‐enrich transport carriers. (B) Molecular details of the association between SNX5 and the cytosolic tail of cargoes such as CI‐MPR and SEMA4C possessing a ФxΩxФ(x)_*n*_Ф consensus motif that folds into a beta harping structure (Ф = hydrophobic and Ω = aromatic sidechains). The first β‐strand always consists of a highly hydrophobic sequence, ^2349^VSYKYS^2354^ in CI‐MPR and ^734^VGYYYS^739^ in SEMA4C. The key side‐chains of SNX5 that mediate binding include Tyr132, Leu133 and Phe136, with the central tyrosine of the βA forming a stacking interaction with SNX5 Phe136. The binding is further sustained by one hydrophobic side chain from βB that packs in the hydrophobic groove of SNX5. In SEMA4C this residue is Leu743, while in CI‐MPR both Leu2370 or Met2371 can support the binding, suggesting that rather than a specific residue, any properly positioned hydrophobic residue in the βB could sustain the interaction.^48^ (C) Model of the oligomeric assembly of SNX‐BARs into tubular lattices that coordinate the biogenesis of cargo‐ladened transport carriers.

SNX1 and SNX2 are the key membrane‐binding components of the ESCPE‐1. They both display a steady state localization on early and late endosomes, and such localization does not require the partners SNX5/SNX6.^24^, ^28^, ^41^, ^48^ The isolated PX domain of SNX1/SNX2 bind PI(3)P, while full‐length proteins have been reported to also bind PI(3,5)P_2_ and PI(3,4)P_2_.^24^, ^25^, ^41^, ^49^ The BAR domain also facilitates membrane‐association through a series of positively charged residues on the concave surface of the dimer that mediate electrostatic interactions with the lipid bilayer^24^, ^59^ (Figure 2A). Supporting the importance of the PX and BAR domains of SNX1 and SNX2 in the interaction with membrane, mutation of residues that compromise the PI binding site, as well as mutations that disrupt the BAR domain, result in cytosolic localization of the protein.^24^, ^48^

Early studies indicated that SNX5/SNX6/SNX32 displayed phosphoinositide binding activity,^60^, ^61^ but a recent screen indicated that the PX domains of these proteins do not display detectable lipid binding capability.^25^ Consistent with this, crystal structures of the PX domains of SNX5 and SNX32 have established that they lack key residues required for lipid binding. Rather, it appears that the PX domain of these SNX‐BARs is uniquely specialized to bind to the specific sequence motifs present in cytosolic tails of transmembrane cargo.^48^, ^62^, ^63^ Full‐length SNX5/SNX6/SNX32 rely on heterodimeric assembly with SNX1 or SNX2 to associate with endosomal membranes^30^, ^42^, ^48^ (Figure 2A) Accordingly, mutations of the charged residues on the concave face of the SNX5‐BAR domain, or mutation of the residues in the dimerization interface result in impaired membrane association.^64^, ^65^

### 2.2 ESCPE‐1 directly binds cargoes

Several SNXs act as cargo adaptors that directly bind transmembrane proteins to regulate, alone or in concert with multimeric complexes such as Retromer and Retriever, cargo trafficking through the endosomal network.^14^ Among these, SNX27 and SNX17 mediate the endosome‐to‐plasma membrane recycling of a myriad of cargoes by binding PDZ binding motifs and NPxY/NxxY sorting motifs, respectively,^52^, ^66^, ^67^, ^68^, ^69^, ^70^, ^71^ recently reviewed in Ref. [63]. All components of ESCPE‐1 were initially characterized for their abilities to bind a variety of receptors including the epidermal growth factor receptor (EGFR), platelet‐derived growth factor receptor (PDGFR), insulin receptor (INSR), leptin receptor and several members of the transforming growth factor (TGF)‐β receptor family^22^, ^23^, ^72^, ^73^, ^74^ (Table 1). More recently, the proteomic interrogation of SNX‐BAR immunoprecipitates and unbiased labelling of the plasma membrane and TGN have vastly expanded the cohort of receptors that were found to associate with SNX5/SNX6.^42^, ^47^, ^48^, ^51^, ^75^ Furthermore, the use of recombinant binding assays such as isothermal titration calorimetry (ITC) has revealed the direct nature of some of these interactions. Among the cargoes found to directly interact with SNX5/SNX6 are the CI‐MPR, the insulin‐like growth factor‐1 receptor (IGF1R), semaphorin 4C (SEMA4C) and neuropilin‐1 (NRP1)^46^, ^48^, ^51^ (Table 1).

**TABLE 1:** Summary of known ESCPE‐1 cargoes and pathogens reported to hijack them as host factors for infection of human cells.

Receptor	Interaction with ESCPE‐1 subunit	Direct binding confirmed	ESCPE‐1 trafficking route	Implications in pathogen entry			
CI‐MPR	SNX5/6/32^42^, ^46^, ^47^, ^48^	YES^46^, ^48^	Endosome‐to‐TGN^42^, ^46^, ^47^, ^48^	Virus^166^	VZV		
IGF1R	SNX5/6/32^46^, ^47^, ^48^	YES^48^	Endosome‐to‐PM^47^, ^48^	Virus^167^	RSV		
NRP1	SNX5/6/32^42^, ^51^	YES^51^	Endosome‐to‐TGN^51^	Virus^145^, ^158^, ^159^	HTLV‐1	EBV	SARS‐CoV‐2
EGFR	SNX1/2/5/6^22^, ^23^, ^72^, ^74^	YES^22^, ^23^, ^72^, ^74^	Endosome‐to‐PM^74^	Virus^139^	HCMV	HPV	RSV
					HCV	IAV	SARS‐CoV
					EBOV	LASV	PRRSV
					TGEV	JEV	HSV‐1
				Bacteria^139^	*Campylobacter jejuni*		
					*Shigella flexneri*		
					*Salmonella typhimurium*		
					*Chlamydia pneumoniae*		
PDGFR	SNX1/2/5/6^22^, ^23^, ^72^	YES^22^, ^23^, ^72^	TBC	Virus^139^	HCMV	IAV	
				Bacteria^139^	*Chlamydia trachomatis*		
					*Campylobacter jejuni*		
AXL	SNX5/32^42^	NO	TBC	Virus^139^, ^168^	CHIKV	TCRV	YFV
					RRV	LCMV	VACV
					EEEV	EBOV	VSV
					SINV	MARV	SV40
					AMAV	AcMAPV	PICV
					LASV	DENV	WNV
					lentivirus	ZIKV	SARS‐CoV‐2
EPHA2	SNX5/6/32^42^, ^51^	NO	Endosome‐to‐TGN^51^	Virus^139^	KSHV	HCV	
				Bacteria^139^	*Chlamydia trachomatis*		
MET	SNX5/6/32^42^, ^48^, ^51^	NO	Endosome‐to‐TGN + PM^48^, ^51^	Virus^139^	AAV2	IAV	EBOV
				Bacteria^139^	*L. monocytogenes*		
ITGA5	SNX5^42^, ^48^, ^51^	NO	Endosome‐to‐TGN + PM^48^, ^51^	Virus^169^	EBOV	EBV	FMDV
					AAV2/5	HIV‐1	
CD44	SNX5/32^42^, ^51^	NO	Endosome‐to‐TGN^51^	Bacteria^170^	*Group A Streptococcus*		
LRP1	SNX5/6^42^	NO	TBC	Bacteria^171^, ^172^	*Clostridium difficile*		
					*Clostridium perfringens*		

Importantly, structural analysis of the minimal CI‐MPR and SEMA4C peptides bound to the PX domain of SNX5 revealed the molecular basis for these interactions^48^ (Figure 2B). CI‐MPR and SEMA4C bind an evolutionarily conserved extension that is unique to the PX domain of SNX5/SNX6 (this extension is absent in fungal Vps17).^48^ Cargo interaction with the SNX5 PX domain occurs via a bipartite structure, consisting of two antiparallel β‐strands (βA and βB) connected by a variable region (Figure 2B).^48^ The βA of the aforementioned cargoes conforms to a ФxΩxФ consensus motif, where Ф = hydrophobic and Ω = aromatic side‐chains^46^, ^48^ (Figure 2B). Proteomics and bioinformatic studies suggest that dozens of transmembrane proteins contain a putative ESCPE‐1 binding motif, but further work will be required to determine whether these are indeed recognized and recycled by ESCPE‐1.^46^, ^48^, ^51^

### 2.3 Membrane remodeling properties of ESCPE‐1

Upon localisation to membranes, it is thought that the increased concentration of SNX‐BARs serves to promote their oligomerisation into loosely ordered spiral arrays, which result in vesicle‐to‐tubule membrane remodeling^30^, ^76^, ^77^ (Figure 2D). In vitro liposome‐based membrane remodeling assays revealed that both purified SNX1 and SNX2 are able to induce the formation of tubules in a concentration‐dependent fashion, while SNX5 and SNX6 fail to do so.^24^, ^30^, ^77^ Also, overexpression of SNX1 and SNX2 in HeLa cells induces the formation of extended tubules emanating from endosomes.^24^, ^41^, ^42^

The transition between the local membrane deformation induced by SNX‐BAR dimers to the formation of tubular profiles is a cooperative process that involves three mechanisms: (1) adherence of the curved BAR‐domain dimer onto the membrane via co‐incidence detection of membrane features; (2) the insertion of shallow amphipathic helices (AHs) that disrupts local lipid organization imposing further membrane curvature and (3) tip‐to‐tip and side‐to‐side interactions between dimers that organize the formation of an oligomeric tubular lattice that translates the local membrane‐sculpting properties of the single dimer into a global tubular remodeling of the membrane.^30^, ^77^ Interestingly, molecular simulation has estimated that a 30%–40% coverage of BAR proteins on the membrane is sufficient to form a continuous coat that shapes and stabilizes these tubular profiles^78^, ^79^ (Figure 2D).

Cryoelectron tomography has provided structural validation of the tenets of SNX‐BAR domain binding. In the structure of homodimeric membrane‐bound SNX1, electrostatic associations and insertion of an AH facilitate membrane binding and curvature.^77^, ^80^ Moreover, the PX domains of several SNXs contain a membrane insertion loop that further promotes membrane deformation.^81^ Tip‐to‐tip interactions promote the association between adjacent SNX‐BAR dimers.^30^, ^77^, ^80^ Interestingly, SNX1:SNX6 heterodimers exhibited greater irregularity on tubules compared with SNX1:SNX1 homodimers, which may reflect the less extended BAR domain in SNX5/SNX6 and correspond to the lack of tubulating ability of SNX5/SNX6/SNX32 in isolation.^30^, ^77^ Together, through these biophysical properties and the concurrent association with cargo, ESCPE‐1 can sequester cargo into tubular recycling profiles that ultimately undergo scission from the endosome (Figure 2B,C).

### 2.4 Elongation and fission of ESCPE‐1 tubular carriers

**FIGURE 3:**
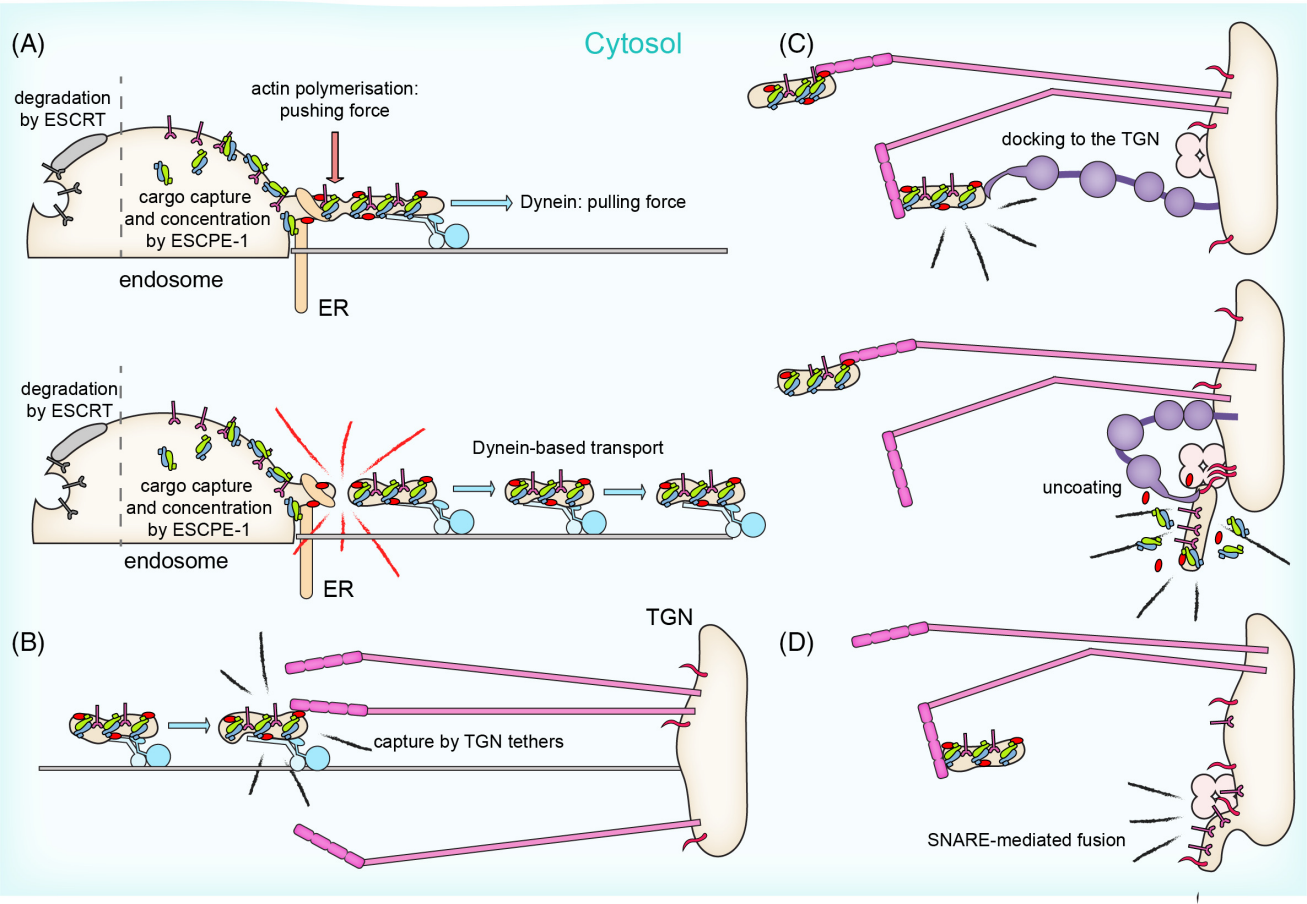
Endosome‐to‐TGN transport of ESCPE‐1 carriers is a multi‐step process that involves several facilitators and regulators. (A) ESCPE‐1 captures and concentrates its cargoes in tubular endosomal subdomains. These tubular profiles, shaped by ESCPE‐1 oligomers, are subjected to the pushing force exercised by actin polymerization (driven by the WASH complex), the pulling force provided by the motor protein dynein, and additional forces including ER membrane contact sites. This leads to the fragmentation into cargo‐enriched tubulovesicular intermediates. (B) These carriers are carried by dynein in proximity of the TGN where they are tethered by TGN‐resident tethers, for example, Golgins. (C) Further TGN‐resident factors ensure the docking and fusion of the carrier to ensure efficient (D) delivery of its cargoes.

Following cargo enrichment, carrier scission is mediated by a complex symphony of biochemical and biophysical forces imposing tension on the tubule. The WASH complex has a primary role in facilitating the extension of the ESCPE‐1 tubular profiles and in regulating their fission through the activation of a localized Arp2/3‐mediated actin nucleation.^19^, ^82^, ^83^ The WASH complex is recruited to ESCPE‐1 tubular domains via the association with the SNX1 interactor RME‐8 thereby coordinating its activity with the membrane remodeling ability of the SNX‐BARs.^84^ The WASH‐dependent formation of branched filamentous actin may also contribute to the fission of the tubular profiles by providing a pushing force to induce membrane tension^19^, ^85^ (Figure 3A). Accordingly, depletion of RME‐8 or the WASH complex gives rise to long membrane tubules extending from endosomes and prevents the correct retrograde trafficking of the ESCPE‐1 cargo CI‐MPR.^82^, ^83^, ^84^, ^86^

More recently, it has been shown that tubular profiles with a BAR‐protein coat can undergo spontaneous friction‐driven scission.^87^ A biophysical model suggests that the elongation force of molecular motors pulling on a coated tubular membrane causes tension between the lipids, leading to pore nucleation and tube scission.^87^ Conceptually, this model could apply to the ESCPE‐1 tubular profiles, the elongation of which is assisted by components of the dynein/dynactin microtubule motor^45^, ^61^, ^88^, ^89^ (Figure 3A). SNX5 and SNX6 interact with p150^glued^,^45^, ^88^ a component of the dynactin complex, which activates the minus‐end directed microtubule motor dynein.^90^ Similarly, SNX4 engages dynein via KIBRA as an intermediate,^32^ and microtubule motor suppression perturbs SNX8 endosomal tubule scission, leading to increased mixing of different SNX‐BARs within the same tubule.^89^ Hence, it is possible that the association with molecular motors could facilitate the extrusion of the endosomal membrane into a tubular profile, promote elongation, and create a longitudinal force that assists the fission (Figure 3A). Consistent with this, ESCPE‐1 tubules have an excessive length upon suppression of p150^glued^, dynein‐1 heavy chain (DHC1) or dynein light intermediate chain 2 (LIC2).^45^, ^89^

Finally, membranes of the ER can control the site and the timing of these fission events by forming contacts with endosomes.^91^, ^92^, ^93^ The formation of ER–endosome contact sites at the neck of a tubular profile may contribute to limit the free diffusion of cargo and could function as a platform for the recruitment of fission machineries^91^, ^92^ (Figure 3A). Coronin‐1C recruits the transmembrane ER protein TMCC1 to endosomal tubules, which plays a role in scission and successful cargo sorting.^94^ Through an interplay with the Arp2/3‐nucleating activity of the WASH complex, Coronin‐1C restricts branched actin to the base of endosomal tubules, defining the site of fission.^95^ The local regulation of phosphoinositides may also be key to ESCPE‐1 tubule scission. From this perspective, a SNX2:VAP‐A/B complex mediates ER‐endosome contact sites to regulate endosome‐localized PI(4)P microdomains, the perturbation of which lead to an exaggerated WASH‐dependent actin polymerization.^96^ In all likelihood, a combination of the above‐mentioned processes, with other emerging fission machineries such as the CROP complex, will provide efficient membrane scission, although how these activities are coordinated and regulated remains to be addressed.^97^

### 2.5 Delivery of ESCPE‐1 carriers to target compartments

Following fission, the ESCPE‐1‐coated tubular carrier couples to microtubule‐associated motors for the long‐range microtubule‐dependent transport to the target compartment. The endosome‐to‐TGN transport of cargo proteins such as the CI‐MPR is achieved through the association of SNX5/SNX6 with the p150^glued^ component of the dynactin complex resulting in the centripetal transport of the post‐endosomal carrier^45^, ^61^, ^88^, ^89^ (Figure 3B).

Similar to the host machineries that sort endosomal cargoes into retrograde recycling carriers, multiple proteins act cooperatively at the TGN to capture, tether and fuse incoming vesicles to complete the trafficking process. Golgins are extended coiled‐coil proteins that associate with Golgi membranes and protrude into the cytosol to form a “tentacular” matrix.^98^, ^99^ Golgins utilize N‐terminal sequences to mediate cargo specificity in the capture of incoming transport carriers throughout the Golgi including retrograde transport vesicles arriving from the endosomal network.^99^, ^100^, ^101^ Golgin‐245 and Golgin‐97, but not the other TGN‐localized Golgins GCC88 and GCC185 captures ESCPE‐1 retrograde carriers that mediate CI‐MPR endosome‐to‐TGN transport.^101^, ^102^ The recognition of ESCPE‐1 vesicles by Golgins might be facilitated by accessory factors, such as the catalytically inactive member of the Rab GTPase‐activating proteins (GAPs) TBC1D23 that was found to associate with Golgin‐245 (and Golgin‐97) to capture retrograde carriers approaching the TGN membranes^103^, ^104^, ^105^ (Figure 3B).

Following engagement of incoming membrane carriers, the precise mechanism that brings them into direct proximity of the TGN membrane is unknown but may be mediated by the Golgin “collapsing” at hinge regions where coiled‐coils are disrupted, or the “hopping” of vesicles between Rab‐binding sites on the Golgin coils to bring them into closer proximity to the membranes.^98^, ^99^ Once the carrier is close enough to the TGN membrane it could be handed over to different factors that regulate the final steps of vesicle tethering and docking, and facilitate carrier fusion with the target membrane. Among these factors are the TGN‐resident Rab6 interacting protein 1, Rab6IP1 (also called DENND5), which interacts with the N‐terminal region of SNX1,^45^, ^106^, ^107^ the SNARE complex containing VAMP3, Syntaxin‐16, Syntaxin‐10 and VTI1a, which is necessary for efficient retrograde transport of the CI‐MPR,^108^ and the TGN‐enriched PI(4)P that facilitate the release of the ESCPE‐1 coat^61^ (Figure 3C).

As ESCPE‐1 also mediates endosome‐to‐plasma membrane recycling, coupling to a plus‐end directed microtubule motor complex(es) is likely to be required for carrier transport toward the cell periphery. However, at the present time the motor protein responsible for the plasma membrane‐directed transport has not been identified. It has been shown that kinesin‐1 is required for the motility of SNX1‐positive endosomes and the organization of SNX1 sub‐domains,^89^ whether the same motor complex also contributes to the peripheral transport of the ESCPE‐1‐decorated cargo‐enriched carriers remains to be investigated.

## 3 ESCPE‐1 IN HOST–PATHOGEN INTERACTION

The catabolic endosomal and autophagic clearance pathways represent an innate cellular defense against invading pathogens. Accordingly, many intracellular pathogens subvert host endosomal sorting pathways to circumvent lysosomal degradation and promote survival and proliferation within cells.^109^ For example, various bacterial pathogens exploit endosomal‐associated machineries to promote the generation of permissive replication niches.^110^ Moreover, many viruses are internalized into the cells through endocytic pathways after binding of host receptors, then rely on endosomal maturation and acidification to trigger their uncoating and genome release, thereby preventing exposure of the genome to nuclease enzymes present in the mature lysosome.^111^ Endosome‐associated machineries such as Retromer, Retriever and the CCC and WASH complexes are key regulators of endosomal biology and hence it does not surprise that they are often subverted by infecting agents for their advantage.^112^, ^113^, ^114^ ESCPE‐1 is no exception and recent work has reappraised the importance of this coat complex in the tug‐of‐war between host and pathogens.

### 3.1 ESCPE‐1 and bacteria

**FIGURE 4:**
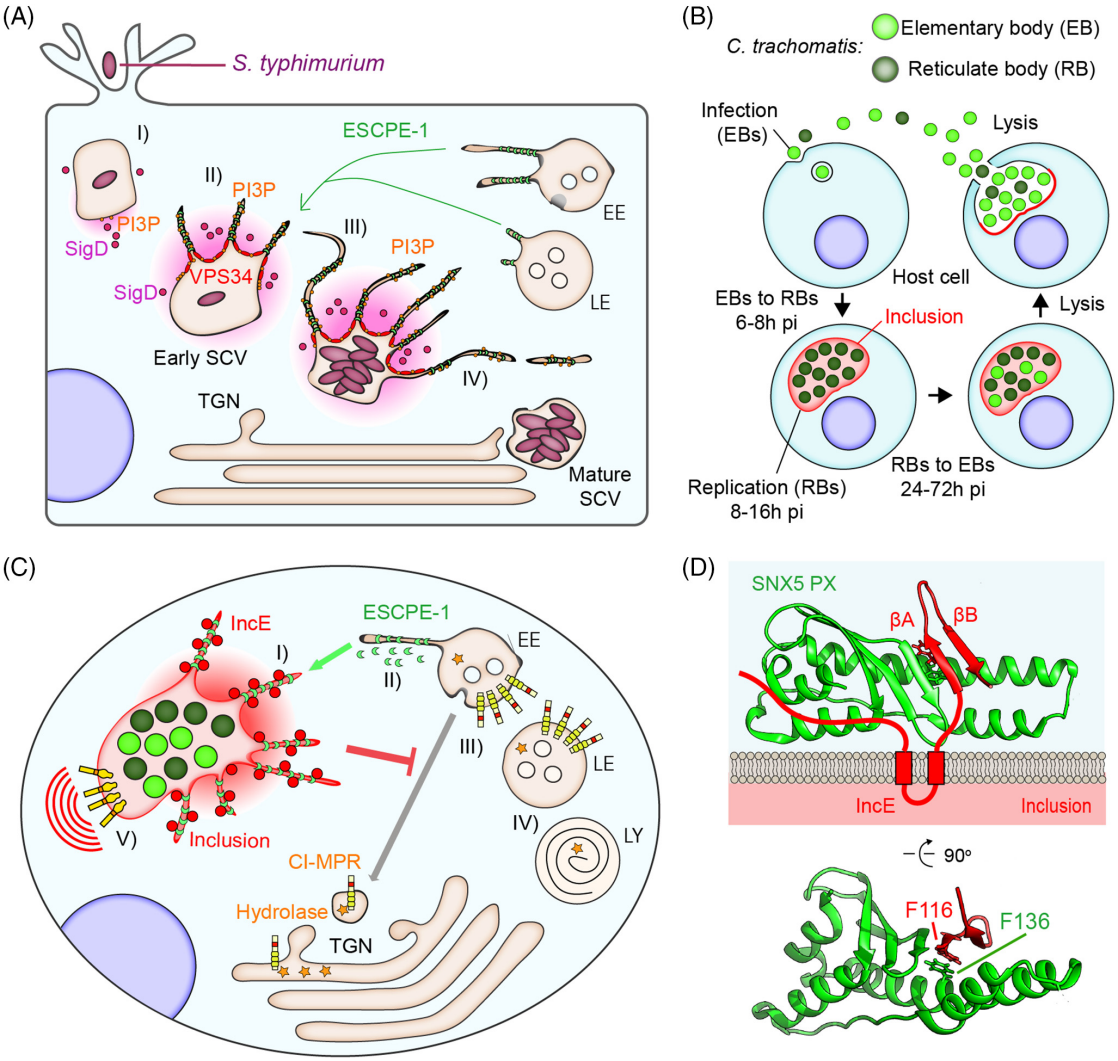
SNX5 in *S. Typhimurium* and *C. trachomatis* infection (A, I) Once *S. Typhimurium* has invaded a cell, it localizes in a membrane‐enclosed compartment, the Salmonella‐containing vacuole (SCV). (II) During an early phase of infection, *S. Typhimurium* releases the inositol polyphosphatase SigD/SopB into the cytosol, which hydrolyses a variety of phosphoinositides facilitates the recruitment of Rab5 and the PI 3‐kinase VPS34. The exaggerated recruitment of VPS34 leads to an elevated increase of PI(3)P on the SCV. (III) The manipulation of phosphoinositide levels drives the enrichment of ESCPE‐1 on to the SCV membrane and triggers the (IV) formation of vacuole‐associated tubules that allow SCV maturation. (B) Scheme of *C. trachomatis* replicative cycle. EBs, elementary bodies; h, hours; pi, post‐infection; RBs, reticulate bodies. (C) Illustration of ESCPE‐1 recruitment by chlamydial IncE and hypothesis of its pathogenic advantages. Once *C. trachomatis* has invaded a cell, it localises in a membrane‐enclosed compartment called the inclusion. (I) At early stages of infection, *C. trachomatis* expresses the virulence factor IncE in the inclusion membrane. (II) IncE directly binds to ESCPE‐1 through SNX5/SNX6 and drives the tubulation of the inclusion membrane. (II) In so doing, IncE prevents ESCPE‐1 from carrying out its cellular functions including the trafficking of the CI‐MPR receptor that plays a role in lysosomal function by delivering newly synthesised hydrolyses to the acidic compartment. (IV) By blocking the CI‐MPR endosome‐to‐TGN cycle, *C. trachomatis* could disrupt lysosomal function and avoid its clearance by the host cell. (V) Furthermore, by altering the association between ESCPE‐1 and a variety of receptor tyrosine kinases, *C. trachomatis* could alter cellular signalling cascades to promote bacterial replication. (D) Molecular basis for the interaction between the extended PX domain of SNX5 and IncE possessing a sequence that folds into a beta harping structure (βA–βB) that mimics that of endogenous ESCPE‐1 cargoes. The key residues forming a stacking interaction between the viral effector and the host protein are highlighted in the structure.

*Salmonella enterica* serovar Typhimurium (*S. Typhimurium*) is one of the best‐known examples of bacteria hijacking ESCPE‐1. This pathogen promotes the recruitment of ESCPE‐1, as well as all the other endosomal SNX‐BARs, to the bacteria‐containing membrane to control the maturation of its intracellular replicative niche, termed the Salmonella Containing Vacuole (SCV)^115^, ^116^ (Figure 4A). *S. Typhimurium* invades cells, and resides within the SCV, which matures in a distinct pathway to that of the endo‐lysosomal network.^116^ During the early stages of infection, *S. Typhimurium* releases its effector SigD/SopB into the cytosol, an inositol polyphosphatase that hydrolyses a variety of PIs and promotes the recruitment of Rab5 and the PI3‐kinase VPS34, overall contributing to the increase of PI(3)P on the SCV^116^, ^117^ (Figure 4A). The manipulation of PI levels drives the enrichment of SNX‐BAR proteins on to the SCV membrane and triggers the formation of vacuole‐associated tubules that allow SCV maturation.^115^, ^116^ The exaggerated ESCPE‐1 and SNX‐BAR‐driven tubulation leads to a faster condensation of the pathogen‐containing membrane that shrinks toward the bacteria until it eventually adheres to the pathogen, forming the SCV^115^, ^116^ (Figure 4A).

The Q fever‐causing bacterium *Coxiella burnetii* requires both Retromer and the ESCPE‐1 to progress in its replication.^118^ Furthermore, *Listeria monocytogenes*, the causative agent of listeriosis, secretes the virulence factor Lmo1656 that binds SNX6 to subvert its function during early stages of oral listeriosis.^119^ The ESCPE‐1 coat complex is also used by toxins en route to different subcellular compartments, including Shiga toxin B‐subunit which requires ESCPE‐1 for efficient endosome‐to‐TGN transport.^120^, ^121^, ^122^

### 3.2 ESCPE‐1 and *Chlamydia trachomatis*

In other examples, ESCPE‐1 acts as a restriction factor during the infectious processes; here pathogens inhibit ESCPE‐1 activity to favour their replication. *Chlamydia trachomatis*, the primary pathogenic agent causing non‐congenital blindness, namely trachoma, has a biphasic life cycle alternating between an infectious spore‐like state termed the elementary body (EB) and an intracellular non‐infectious replicative form called the reticulate body (RB)^123^, ^124^ (Figure 4B). Upon cellular entry, *C. trachomatis* resides and replicates in the inclusion, a membrane‐bound vacuolar compartment that originates from the endo‐lysosomal network.^123^, ^124^ In the protected environment of the inclusion, *C. trachomatis* cycles between infectious EB and replicative RB stages (Figure 4B), orchestrated by the secretion of a cocktail of virulence factors that manipulate the host cell to reconfigure the cytoskeleton and microtubule‐based motors, extract nutrients, avoid the host defenses and inhibit catabolic processes.^123^, ^124^ Among these virulence factors is the transmembrane effector protein IncE, which is incorporated by a type III secretion system into the encapsulating inclusion membrane^125^, ^126^ (Figure 4C). IncE harbours a ФxΩxФ‐like beta hairpin structure which directly binds the cargo‐selective PX domains of SNX5 and SNX6 to recruit ESCPE‐1 to the inclusion membrane^62^, ^75^, ^127^ (Figure 4D).

Suppression of ESCPE‐1 expression enhances EB progeny production in infected cells, suggesting that the interaction prevents a restrictive function performed by ESCPE‐1.^125^, ^126^ IncE has a low micromolar affinity for SNX5/SNX6 PX domain and this results in the out‐competition of ESCPE‐1 interaction with its endogenous cargoes, suggesting that multiple crucial cellular trafficking processes could be reconfigured by IncE expression.^48^, ^62^, ^75^, ^127^ Among the cargoes that are outcompeted by IncE, CI‐MPR plays a role in lysosomal function by delivering newly synthesized hydrolyses to the acidic compartment.^128^ Hence, the IncE‐induced re‐localisation of ESCPE‐1 could lead to disrupted lysosomal function by blocking the CI‐MPR endosome‐to‐TGN cycle (Figure 4C). Moreover, missorting of the CI‐MPR also causes mistrafficking of the Niemann–Pick Disease Type C2 protein (NPC2), leading to a pronounced accumulation of cholesterol in the endo‐lysosomal membranes^129^ (Figure 4C). Because *C. trachomatis* obtains eukaryotic cholesterol from the host cell and the inclusion is tightly associated with the endocytic membranes, the disruption of ESCPE‐1 mediated trafficking could facilitate the process of cholesterol accumulation on the inclusion.^124^, ^130^ Finally, *C. trachomatis* secures its survival within the host cell by modulating antiapoptotic and prosurvival signalling pathways.^124^, ^131^ Because ESCPE‐1 interacts with several transmembrane signalling receptors, including EPHA2 and MET, it is tempting to speculate that the secretion of IncE at early stages of infection results in altered cellular signalling cascades that play in favour of bacterial replication^42^, ^48^, ^51^ (Figure 4C).

### 3.3 ESCPE‐1 and viruses

SNX5 has emerged as a critical host factor for the replication of a number of alphaviruses including Sindbis (SINV), Mayaro (MAYV) and Chikungunya (CHIKV) viruses, although the molecular bases of these processes are yet to be uncovered.^132^ SNX5 also promotes the infection of human cytomegalovirus (HCMV) through direct interaction with its tegument protein ppUL35; here the diversion of SNX5 results in a defective retrograde transport of CI‐MPR that might facilitate virus replication.^133^ Human respiratory syncytial virus (HRSV) assembles its genetic material with newly synthetized fusion protein F, and structural proteins M and N at the plasma membrane to form progeny virions.^134^ HRSV N and M mainly localize in viral inclusion bodies, which are the replicative niches of HRSV, but are also found in the TGN, from where they are exported to the PM for the assembly of HRSV virions.^135^ It has been reported that SNX1/SNX2 plays an important role for the trafficking of M and N proteins to the assembly sites.^135^ Furthermore, N interacts with SNX2 and might recruit this host protein to viral inclusion bodies and filamentous assembly sites at the PM.^135^ Consistently, suppression of SNX1 and SNX2 led to a reduction of viral proteins, size of inclusion bodies, inhibition of virus‐induced cell–cell fusion, and progeny production.^135^ Of note, the subversion of ESCPE‐1 by viruses has been reported also outside of the animal kingdom. ESCPE‐1 promotes the formation and stabilization of the tomato bushy stunt virus (TBSV) membrane‐bound viral replicase complex in plants cells, and in the surrogate host yeast.^136^, ^137^

### 3.4 ESCPE‐1 and antiviral xenophagy

On the other hand, SNX5 also plays a role in cellular immunity against some viruses, thereby protecting cells and organisms from viral infections.^65^, ^138^ Using a genome‐wide short interfering RNA screen, SNX5 and SNX32 were identified as essential factors promoting virus‐induced, but not basal or stress‐induced, autophagy (specifically called xenophagy).^65^ Indeed, suppression of SNX5 (or SNX32) in HeLa cells enhanced cellular susceptibility to infection with a variety of viruses including SINV, herpes simplex virus type 1 with a deletion that prevents host autophagy inhibition (HSV‐1ΔBBD), Zika virus (ZIKV), West Nile virus (WNV), CHIKV, Poliovirus (PVR), Coxsackievirus B3 (CVB3) and Influenza A virus (IAV).^65^ Consistently, it was observed that *Snx5* knockout mice displayed higher lethality after infection with several of these human viruses (SINV, WNV, HSV‐1 ΔBBD and CHIKV).^65^

**FIGURE 5:**
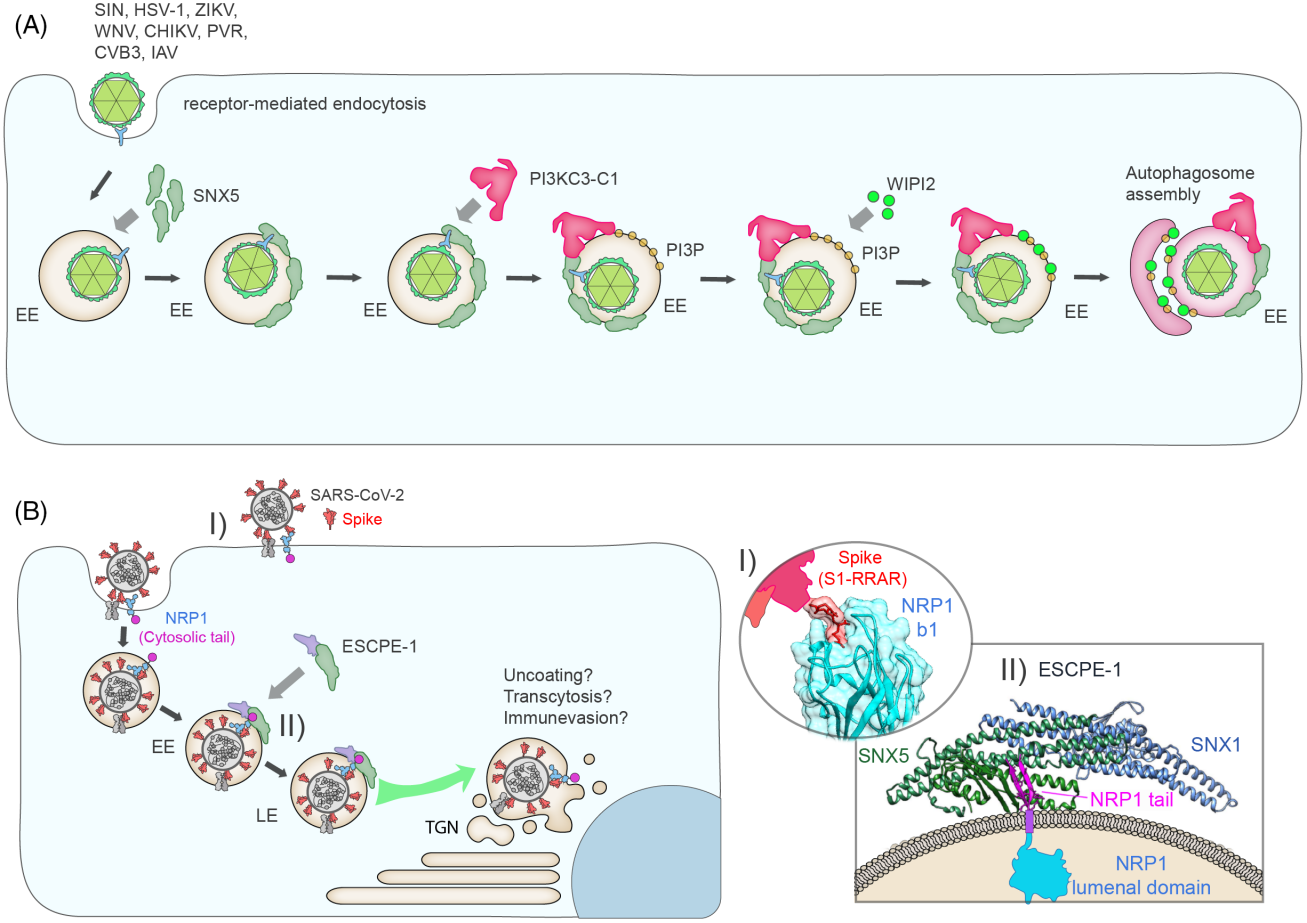
Roles for ESCPE‐1 in viral infection (A) Viruses are internalised into the host cell via multiple pathways including receptor‐mediated endocytosis. SNX5 can facilitate the clearance of several internalized viruses through the process of viral‐induced autophagy. In this case, the virus‐containing endosomes are recognized by SNX5, which interacts with Beclin‐1 and ATG14‐containing class III phosphatidylinotol‐3‐kinase (PI3KC3) complex 1 (PI3KC3‐C1). The SNX5‐PI3KC3‐C1 complex promotes the generation of PI(3)3P on virus‐containing endosomes and facilitates the recruitment of the PI3P‐binding protein WIPI2. This results in the initiation of autophagosome assembly and viral clearance. (B) Model for ESCPE‐1 dependent trafficking of internalised SARS‐CoV‐2. (I) SARS‐CoV‐2 binds NRP1 at the cell surface through direct interaction between the CendR motif of S1 and the extracellular NRP1 b1 domain. (II) Once internalised into the endosomal network, the cytosolic tail of NRP1 directly binds SNX5/SNX6, subverting ESCPE‐1 coat assembly, membrane deformation and coupling to the cytoskeleton. The recruitment of ESCPE‐1 could facilitate membrane fusion or trafficking of SARS‐CoV‐2 and the Spike protein.

The mechanism by which SNX5 facilitates xenophagy depends on its ability to interact with Beclin‐1 and ATG14‐containing class III phosphatidylinotol‐3‐kinase (PI3KC3) complex 1 (PI3KC3‐C1).^65^ This interaction promotes the generation of PI(3)P on virus‐containing endosomes and drives the recruitment of the PI(3)P‐binding protein WIPI2 that initiate the autophagosome assembly^65^ (Figure 5A). An outstanding question relates to how luminal viruses stimulate the ESCPE‐1‐PI3KC3 axis on the cytoplasmic face of endosomes? Presently, we can only speculate on likely mechanisms. SNX5 associates with integral receptor tyrosine kinases that serve as viral entry factors,^139^ such as AXL (e.g., ZIKV), IGF1 receptor (e.g., HRSV), EPHA2 (e.g., Epstein–Barr virus, EBV), MET (e.g., adeno‐associated viruses) and EGFR (e.g., IAV)^42^, ^48^, ^140^, ^141^ (Table 1). Viral‐induced enrichment of these membrane proteins may provide a compartment “signature” for recruiting the SNX5‐PI3KC3 axis to virus‐containing endosomes (Figure 5A). Additional mechanisms, such as viral induction of local membrane curvature (sensed by the SNX5 BAR domain) and/or virally encoded membrane penetrating peptides that present ESCPE‐1 binding motifs to the cytoplasm (as in the case of the human papillomavirus, HPV) may serve to add complexity to the recruitment signature of viral containing endosomes.^142^ It also remains to be addressed whether the SNX1 and SNX2 subunits of ESCPE‐1 contribute to this these processes.

### 3.5 ESCPE‐1 and SARS‐CoV‐2

In addition to detecting the presence of transmembrane cargo as a trigger for virus‐induced xenophagy, ESCPE‐1 activity may also influence the spatiotemporal trafficking of viral receptors through the endosomal network to the cell surface or TGN, thereby influencing infection efficiency and subsequent trafficking routes exploited by viruses. Severe acute respiratory syndrome coronavirus 2 (SARS‐CoV‐2), the causative pathogen of the Coronavirus Disease 2019 (COVID‐19) pandemic, engages its primary receptor angiotensin‐converting enzyme 2 (ACE2) at the cell surface to mediate infection.^143^, ^144^ Additionally, recent work characterized NRP1 as a host factor for SARS‐CoV‐2.^145^, ^146^ NRP1 acts as a multifunctional co‐receptor that orchestrates ligand binding to signalling receptors, a subset of which harbour a C‐terminal polybasic sequence termed the C‐end rule (CendR).^147^, ^148^ The SARS‐CoV‐2 Spike (S) protein exhibits molecular mimicry through the exposure of a CendR motif in the S1 subunit following proteolytic cleavage to exploit this binding modality and enhance infection of human cells^145^, ^146^, ^149^, ^150^, ^151^ (Figure 5B).

A proximity proteomics screen of TGN‐resident proteins recently identified NRP1 as a cargo depleted from the TGN upon ESCPE‐1 suppression.^51^ Intriguingly, NRP1 recycling to the cell surface was unaffected in ESCPE‐1 KO cells, suggesting specificity for the retrograde pathway. Immunoprecipitation of SNX5/SNX6 revealed co‐isolation of both NRP1 and SARS‐CoV‐2 S through a mechanism dependent on the ability of the PX domain to specifically engage cargo.^51^ ESCPE‐1 KO reduced the rate of internalized CendR‐coated nanoparticle trafficking to the perinuclear region, indicating the ESCPE‐1 activity can directly influence the destination of NRP1‐associated ligands (Figure 5B). Moreover, ESCPE‐1 KO cells are less permissive to SARS‐CoV‐2 infection, suggesting that the spatiotemporal sorting of NRP1 by ESCPE‐1 influences the infection process.^51^

## 4 CONCLUDING REMARKS AND FUTURE PERSPECTIVES

In conclusion, in recent years our understanding of ESCPE‐1‐mediated cargo recycling has been greatly expanded. Furthermore, the role of ESCPE‐1 function in bacterial and viral infections is becoming clearer and could be of pivotal importance in uncovering novel pathways to target for innovative therapeutic approaches. For example, small molecules that inhibit SNX1‐dependent endosomal tubulation and retrograde trafficking of Shiga toxins were recently identified.^152^ We hope that this work could encourage the exploration of ESCPE‐1 role in host–pathogen interaction and promote the identification of molecular interventions to target such interactions for reduced infection.

### 4.1 Deciphering the dimerisation code

An interesting question is the biological significance of why there are eight possible different dimeric combinations within ESCPE‐1. The consensus assumption is that the paralogues SNX1/SNX2 have redundant functions, and this is mainly based on the observation that SNX1‐null mice or SNX2‐null mice are viable and fertile while mice lacking both SNX1 and SNX2 die during mid‐gestation.^38^, ^39^ However, the SNX1‐null mice harbouring a single copy of SNX2 do not show any noticeable difference compared with the wild type litter mates, whereas the SNX2‐null mice harbouring a single copy of SNX1 showed partial embryonic lethality (~40%) and the offspring that survived have signs of growth retardation.^38^, ^39^ These findings suggest that the function of SNX heterodimers may not be fully equivalent.^38^, ^39^ It is tempting to speculate that the variety of ESCPE‐1 heterodimers allows the recruitment of different subsets of accessory proteins and/or points of post‐translational modification that serve to fine‐tune the regulation of cargo sorting and trafficking; furthermore, it is possible that different heterodimeric combinations are required for the recycling of specific pools of cargo proteins. From an evolutionary point of view, it is possible that diversification of ESCPE‐1 functions might have been required to regulate the complexity of receptor trafficking in more complex polarized and associative organisms. Further molecular phylogenetic analysis and biochemical validations will be required to test some of these hypotheses.

### 4.2 Higher order integration of ESCPE‐1 with additional cargo adaptors

Future work will also be required to elucidate the overall architecture of coats consisting of ESCPE‐1 heterodimers and how transmembrane proteins package in these tubular lattices. Given recent data, it remains to be established how additional cargo sorting machineries, such as SNX27‐Retromer, and its cargoes integrate into these structures. SNX1 and SNX2 (but also SNX4, SNX7, SNX8 and SNX30) possess unstructured N‐terminal regions of varying length and of uncharacterised function. Having recently established that the N‐terminal regions of SNX1 and SNX2 directly interact with SNX27,^53^, ^54^, ^55^ it will be interesting to investigate whether these domains are mediating a broader set of protein–protein interactions. Spatially restricted biotinylation of the cell surface and TGN revealed cohorts of transmembrane proteins dependent on ESCPE‐1 for their sorting.^48^, ^51^ While many of these cargoes contain ФxΩxФ(x)_*n*_Ф consensus motifs and thus likely represent canonical ESCPE‐1 cargoes, others appear not to. The process of identification of these additional cargoes and their inclusion into ESCPE‐1 tubules likely reflects this crosstalk between endosomal sorting complexes. Interestingly, this low‐complexity N‐terminal region within the SNX8 homologue Mvp1 has been suggested to stabilize soluble Mvp1 in a tetrameric form, with release of the N‐termini unmasking the PX and BAR domains and thereby releasing functional dimers that can associate with membranes.^153^ It will be interesting to explore whether a similar mechanism also accounts for the stabilisation of cytosolic ESCPE‐1 and other SNX‐BARs complexes in mammalian cells.

### 4.3 Post‐translation regulation of ESCPE‐1 sorting

A fascinating open question is whether posttranslational modification spatiotemporally regulates ESCPE‐1 sorting. This has been established for SNX27‐Retromer cargoes, whereby phosphorylation of residues within the minimal C‐terminal PDZ binding motif negatively impairs SNX27 association, but phosphorylation of residues preceding the minimal motif can dramatically enhance affinity for the SNX27 PDZ domain.^71^ At present, the established consensus sequences for ESCPE‐1 binding possess one or more tyrosine residues that are key for the interaction with the hydrophobic pocket of SNX5/6 PX domain.^48^ It is, therefore, conceivable that tyrosine phosphorylation may prevent ESCPE‐1 binding by introducing negative charge into the otherwise hydrophobic consensus sequence. Masking of the ESCPE‐1 motif through phosphorylation may therefore prevent tubular‐based recycling or re‐direct cargo proteins to different destinations. In addition to post‐translational modification of cargo tail sequences, it is also conceivable that ESCPE‐1 itself is subjected to similar spatiotemporal control. The S226 residue of SNX5 undergoes phosphorylation to regulate its propensity to dimerise with SNX1 and SNX2.^64^ ESCPE‐1 cargoes are predominantly sorted to the plasma membrane or TGN, but how this directionality is conferred remains unclear. It is tempting to speculate that post‐translational modifications of ESCPE‐1 and/or its cargo may spatiotemporally regulate its membrane recruitment and cargo sensing, and in turn dictate which endocytic recycling route cargo may follow. This fine‐tuning of ESCPE‐1 activity, its differences across tissues, and its subversion during infection may represent significant avenues of future research.

### 4.4 ESCPE‐1 and NRP1, synergistic viral host factors?

The NRP1:ESCPE‐1 interaction could conceivably influence SARS‐CoV‐2 infection through a number of possible mechanisms. One possibility could lie in the direct association of ESCPE‐1 with the dynein/dynactin complex.^45^, ^88^ NRP1 clustering on the endosomal membrane following the engagement and internalization of SARS‐CoV‐2 may be sensed by ESCPE‐1, thereby coupling the virus:NRP1 complex through to the cytoskeleton. This could conceivably stimulate minus‐end directed trafficking of virus‐containing compartments or could alternatively generate a biophysical pulling force that aids the membrane fusion process. The interferon‐inducible transmembrane protein (IFITM) family has been reported to play a restrictive role in infection of a range of viruses including SARS‐CoV‐2, possibly through modulating the fluidity of endosomal membranes and preventing the successful formation of fusion pores.^154^, ^155^, ^156^ Motor‐driven cytoskeletal forces may therefore produce a counterforce that destabilizes the membrane to aid fusion.^87^ Alternatively, the ESCPE‐1:NRP1 interaction may serve to traffic the Spike protein alone following viral fusion with possible implications for Spike‐driven cell–cell fusion events. Another fascinating hypothesis is that ESCPE‐1 could facilitate the unconventional endocytosis of CendR ligands, including viruses. A preprint has established that SNX2 and the WASH complex mediates a unique type of micropinocytosis, named WASHme, that plays a role in the entry of HPV.^157^ Whether NRP1 and other ESCPE‐1 cargo receptors are involved in WASHme‐mediated entry of viruses remains to be established.

The observation that ESCPE‐1 plays a role in the infection of SARS‐CoV‐2 raises several interesting questions.^51^, ^145^ Epstein–Barr Virus (EBV) and Human T‐Cell Lymphotropic Virus Type 1 (HTLV‐1) have also been shown to use NRP1 for entry and infection.^158^, ^159^ It is, therefore, tempting to speculate that the role of ESCPE‐1 in SARS‐CoV‐2 infection could be extended to other NRP1‐dependent viruses. Given that ESCPE‐1 also regulates the sorting of a wider array of viral receptors, including AXL, IGF1R, EPHA2, MET, EGFR, this effect may also apply to multiple distinct microbial pathogens that rely on these receptors for infection (Table 1). Future studies aiming to modulate or inhibit this pathogenic subversion of ESCPE‐1 cargo sorting, or SNX5‐driven initiation of xenophagy, may provide insights into how to manipulate this pathway to block intracellular pathogen survival.

### 4.5 Other ESCPE complexes

Interestingly, recent work seems to suggest that endosomal‐SNX‐BARs can assemble into dimeric sorting complexes outside of the conventional dimerization pairing. This is the case for the Recycler complex, composed of SNX4:SNX5 heterodimers and SNX17, that plays an essential role in autophagosomal components recycling.^160^ With limited evidence for a functional role of SNX4:SNX7 and SNX4:SNX30 heterodimers, and SNX8 homodimers in direct sorting of endosomal cargoes, future work will be required to dissect the molecular basis for how these heterodimeric SNX‐BAR assemblies participate to the process of cargo recycling, and how this contributes to cellular pathways for host‐pathogen interaction. SNX4 and SNX8 have been linked to the retrograde trafficking of the ricin toxin and Shiga toxin subunit B (STxB) toxins respectively, although mechanistic details of endogenous cargo recycling through this route remain unclear.^122^, ^161^ Interestingly, SNX8 has been reported to be part of a host defense mechanism against *Listeria monocytogenes* and a regulator of innate cellular responses to viruses.^162^, ^163^, ^164^ Clearly, there is still a lot to learn about the cellular functions of endosomal SNX‐BAR proteins, and how their role is subverted by pathogens for infection.

## CONFLICT OF INTEREST STATEMENT

The authors declare no competing financial interests.

5

### PEER REVIEW

The peer review history for this article is available at https://www.webofscience.com/api/gateway/wos/peer-review/10.1111/tra.12885.

## Data Availability

Data sharing not applicable to this article as no datasets were generated or analysed during the current study.
